# Influenza in Malaysian adult patients hospitalized with community-acquired pneumonia, acute exacerbation of chronic obstructive pulmonary disease or asthma: a multicenter, active surveillance study

**DOI:** 10.1186/s12879-021-06360-9

**Published:** 2021-07-05

**Authors:** Yong Kek Pang, Ahmad Izuanuddin Ismail, Yoke Fun Chan, Adelina Cheong, Yoong Min Chong, Paras Doshi, Joanne Zhi Han Lau, Jean Khor, Lilian Phei Lian Wang, Chee Loon Leong, Aisya Natasya Musa, Kee Sing Ng, Mau Ern Poh, I-Ching Sam, Jiunn Liang Tan, Mohd Arif Mohd Zim, Anne-Frieda Taurel

**Affiliations:** 1 Department of Medicine, University Malaya Medical Centre, 59100 Kuala Lumpur, Malaysia; 2 Department of Medicine, Faculty of Medicine, Universiti Teknologi Mara, Selayang Campus, Jalan Prima Selayang, Batu Caves, Selangor Malaysia; 3 Department of Medical Microbiology, Faculty of Medicine, University Malaya, 50603 Kuala Lumpur, Malaysia; 4 Medical Department, Sanofi Pasteur, Plaza 33, 46200 Petaling Jaya, Selangor Malaysia; 5 Department of Medicine, Kuala Lumpur General Hospital, Jalan Pahang, 50586 Kuala Lumpur, Wilayah Persekutuan Kuala Lumpur Malaysia; 6 Vaccine Epidemiology and Modeling Department, Sanofi Pasteur, Singapore, Singapore

**Keywords:** Influenza, Human, Hospitalization, Adults, Epidemiology, Influenza-like illness, Malaysia, Outcomes

## Abstract

**Background:**

Available data on influenza burden across Southeast Asia are largely limited to pediatric populations, with inconsistent findings.

**Methods:**

We conducted a multicenter, hospital-based active surveillance study of adults in Malaysia with community-acquired pneumonia (CAP), acute exacerbation of chronic obstructive pulmonary disease (AECOPD) and acute exacerbation of asthma (AEBA), who had influenza-like illness ≤10 days before hospitalization. We estimated the rate of laboratory-confirmed influenza and associated complications over 13 months (July 2018–August 2019) and described the distribution of causative influenza strains. We evaluated predictors of laboratory-confirmed influenza and severe clinical outcomes using multivariate analysis.

**Results:**

Of 1106 included patients, 114 (10.3%) were influenza-positive; most were influenza A (85.1%), with A/H1N1pdm09 being the predominant circulating strain during the study following a shift from A/H3N2 from January–February 2019 onwards. In multivariate analyses, an absence of comorbidities (none versus any comorbidity [OR (95%CI), 0.565 (0.329–0.970)], *p* = 0.038) and of dyspnea (0.544 (0.341–0.868)], *p* = 0.011) were associated with increased risk of influenza positivity. Overall, 184/1106 (16.6%) patients were admitted to intensive care or high-dependency units (ICU/HDU) (13.2% were influenza positive) and 26/1106 (2.4%) died (2.6% were influenza positive). Males were more likely to have a severe outcome (ICU/HDU admission or death).

**Conclusions:**

Influenza was a significant contributor to hospitalizations associated with CAP, AECOPD and AEBA. However, it was not associated with ICU/HDU admission in this population.

Study registration, NMRR ID: NMRR-17-889-35,174.

**Supplementary Information:**

The online version contains supplementary material available at 10.1186/s12879-021-06360-9.

## Introduction

Influenza is associated with substantial disease burden worldwide, with estimated annual attack rates of 5–10% in adults and 20–30% in children [1]. While most people recover from mild influenza illness within 2 weeks, some individuals suffer from severe illness and complications that may lead to hospitalization and death. Very young age (< 5 years), old age (≥65 years) and underlying chronic respiratory illness are risk factors for severe influenza outcomes (hospitalization, admission to intensive care unit, and death) [2, 3]. Using modeling methods, annual epidemics worldwide were estimated to result in 3–5 million cases of severe illness, and 290,000–650,000 respiratory deaths in 2015 [4, 5].

Southeast Asia was estimated to have one of the highest influenza-associated mortality rates (3.5–9.2 per 100,000 individuals), along with sub-Saharan Africa (2.8–16.5 per 100,000 individuals) [5]. However, these estimates were based on data collected from 33 contributing countries (two in Southeast Asia) extrapolated to countries that had limited to no information available from vital records and viral surveillance making these results reliant on extrapolation [5]. Prior published evidence on the influenza disease burden in Southeast Asia is largely focused on pediatric populations, with inconsistent findings [6, 7].

Improved estimates of disease burden in Southeast Asia are needed, particularly in low- and middle-income countries, to inform strategies for influenza control and resource allocation. Efforts have been made over the last two decades to expand surveillance across the region, with national surveillance systems for influenza-like illness (ILI) and severe acute respiratory infection (SARI) set up in Indonesia (in collaboration with the USA Centers for Disease Control and Prevention) since 2006, the Philippines since 2004 and Malaysia since 2003. In addition, sentinel sites with weekly status reports have also been set up in Taiwan, Thailand and Vietnam. However, the extent and type of influenza surveillance vary across the region, with limited or no coverage in some areas [8]. In Malaysia, influenza is observed year-round [6]; and while it is not a notifiable disease in Malaysia, limited data on cases have been collected (age and site of origin) through the surveillance system since 2003. However, variable knowledge and misconceptions with regard to influenza, including a perceived low circulation of the virus and low severity of the disease, have been demonstrated [9], and may impact the quality of data reporting and analysis. A study conducted in 2010 by the Institute for Medical Research Malaysia showed that only 0.2% of ILI cases included in the study (*n* = 878) had been correctly diagnosed by healthcare providers and that influenza was generally not perceived as a priority disease; the highest ILI consultation rates in the study were recorded among children and youths aged ≤19 years [10].

We conducted a multicenter hospital-based active surveillance study of influenza cases in adult patients with community-acquired pneumonia (CAP), acute exacerbation of chronic obstructive pulmonary disease (AECOPD) and acute exacerbation of asthma (AEBA), to provide insight into the influenza-related severe disease burden in Malaysian adults. We report the rate of laboratory-confirmed influenza and associated complications, and the distribution of causative influenza strains among these cases. We also evaluated predictors of laboratory-confirmed influenza and severe influenza-related outcomes in this population using multivariate logistic regression analysis.

## Methods

### Study design and patients

This was a prospective epidemiological active surveillance study, adapted from the Global Influenza Hospital Network protocol [11], conducted in three hospitals located in the Klang Valley area in Malaysia (an urban conurbation centered around the capital, Kuala Lumpur) over a one-year period, from July 2018 to August 2019. The following sites participated in this study: University Malaya Medical Centre (UMMC), Lembah Pantai, Wilayah Persekutuan; Kuala Lumpur General Hospital, Titiwangsa, Wilayah Persekutuan; and Selayang Hospital (Universiti Teknologi Mara; UiTM), Gombak, Selangor Darul Ehsan. Patients aged ≥18 years hospitalized in the preceding 24–72 h with CAP, AECOPD or AEBA and who had onset of ILI ≤10 days prior to admission were eligible for inclusion. We defined ILI based on a combination of case definitions previously established by the World Health Organization (WHO) and the European Centre for Disease Prevention and Control (ECDC) to maximize sensitivity and specificity [12, 13] with at least one of four systemic symptoms (fever [≥38 °C], headache, myalgia, arthralgia or malaise) in addition to at least one of three respiratory symptoms (cough, sore throat or shortness of breath). Institutionalized patients and those hospitalized in the 30 days prior to the study were excluded. We defined a severe outcome as ICU/HDU admission or in hospital death.

Participant data (age, gender, body mass index (BMI), smoking status, number and type of comorbidities, socio-professional category, diagnosis at admission identified by ICD-9/ICD-10 admission codes [CAP, AECOPD or AEBA], ILI [systemic and respiratory symptoms], health history [number of hospitalizations in the last 12 months; number of outpatient physician consultations in the last 3 months; presence of past confirmed influenza in the previous year] and influenza vaccination status [within the past 12 months and more than 14 days before onset of ILI symptoms]) were collected by completion of a questionnaire by the study nurse/physician through face-to-face interview with the patient, supplemented with available patient clinical records. Comorbidities that involved organ dysfunction, including pre-existing COPD and asthma, were considered to be ‘significant comorbidities’. Socio-professional categories are defined in Additional file 1 and were grouped as follows [14]: high (including managers, executives, self-employed individuals in professions requiring a graduate or post-graduate degree; technicians, artists, athletes, administrative employees/professionals, personal services; security), middle (skilled and semi-skilled manual workers), low (unskilled workers) and unclassifiable. Nasopharyngeal and/or oropharyngeal swabs were collected from each patient and stored at − 80 °C. Swabs were transported on a monthly basis on dry ice to the testing laboratory at the University of Malaya for detection of influenza viruses.

The study protocol and amendments were approved by the Medical Research and Ethics Committee from the Malaysian Ministry of Health (reference KKM/NIHSEC/P17–852), the Medical Research Ethics Committee, University Malaya Medical Centre (ID NO: 2017465126) and the Universiti Teknologi Mara Research Ethics Committee (reference 600-IRMI (5/1/6) and the study was conducted in accordance with Good Clinical Practice and Good Epidemiological Practice guidelines. All included individuals were provided information on the study and signed informed consent forms before any study procedures were performed.

### Laboratory procedures

#### Evaluation and validation of one-step duplex RT-qPCR assay

Clinical influenza isolates were used to synthesize in vitro transcribed RNA controls. A two-step RT-PCR was used and the amplified product was ligated into pJET1.2 (blunt-end cloning vector) using the CloneJET PCR cloning kit (Thermo Fisher Scientific, USA). Cloning was performed in XL-10 gold *Escherichia coli*. A single colony was picked and amplified using the 5′ T7 promoter sequence as the forward primer, with the corresponding RT-qPCR reverse primer for each signature. Amplification products were transcribed using a MEGAshortscript T7 transcription kit (Invitrogen/Life Technologies, USA). The RNA transcripts were purified using MEGAclear transcription clean-up kit (Invitrogen/Life Technologies) and quantified by Epoch Microplate spectrophotometer (BioTek, USA). All transcript dilutions were carried out in nuclease-free water.

#### Extraction of viral RNA

Viral RNA was extracted from 140 μL of each clinical specimen with a QIAcube instrument using the QIAamp viral RNA mini kit (QIAGEN, USA) as per manufacturer’s protocols. RNA was eluted in a final volume of 40 μL and stored at − 80 °C until use.

#### One-step duplex RT-qPCR assay for influenza detection and subtyping

Three different duplex RT-qPCR assays (influenza A and B virus, A/H1pdm and A/H3 subtyping, and B/Yamagata and B/Victoria lineages) were performed according to WHO guidelines [15], with minor modifications. Each sample was first tested with the influenza diagnostic assay, detecting the matrix protein (M) gene of influenza A and the hemagglutinin (HA) gene of influenza B simultaneously. Influenza A-positive samples were further subtyped as H1pdm09 or H3 viruses, and influenza B-positive samples further subtyped to distinguish B/Yamagata and B/Victoria lineages, using the HA gene as the target region. Primer and probe sequences are shown in Additional file 2. Briefly, duplex RT-qPCR assay was performed in a reaction consisting of 4× Taqman Fast Virus 1-step master mix (Applied Biosystems, USA), forward primer, reverse primer, probes and 2.5 μL of RNA template. The reaction was diluted in PCR-grade water to a total reaction volume of 10 μL. Positive and non-template controls were included in each run. The StepOnePlus real-time PCR system (Applied Biosystems) was used for amplification. The thermocycling conditions were: reverse transcription at 50 °C for 5 min and 95 °C for 20 s, followed by 40 cycles of 95 °C for 3 s and 60 °C for 30 s. A reaction with a cycle threshold (Ct) value ≤38 was considered positive. Influenza cases with negative results for subtyping were considered ‘untyped’.

#### Sequencing

Sequencing was performed for A/H1N1pdm09 and A/H3N2-positive samples with subtype Ct values ≤30. HA genes were amplified as overlapping halves using one-step RT-PCR using WHO-recommended primer sets shown in shown in Additional file 2 [15]. Briefly, 5 μL of RNA template was amplified by adding 0.4 μM of forward and reverse primer, 2× MyTaq One-Step mix (Bioline, UK), reverse transcriptase, Ribosafe RNase inhibitor and DEPC-water in a 50 μL mixture. For products > 1 kb amplicon, the RT-PCR reaction was performed at 48 °C for 40 min and 95 °C for 1 min for reverse transcription, followed by 40 cycles of 95 °C for 10 s, 60 °C for 10 s and 72 °C for 30 s using an Applied Biosystems Veriti Thermal Cycler (Thermo Fisher Scientific). For amplicons < 1 kb, the reverse transcription was performed at 45 °C for 20 min. PCR products were visualized by 1% agarose gel electrophoresis and outsourced for sequencing (First BASE Laboratories, Malaysia).

#### Phylogenetic analysis

Chromatograms were edited with ChromasPro 2.1.8 (Technelysium, Australia) and contigs were aligned using Geneious Prime 2019 (Biomatters, New Zealand) with reference and other influenza strain HA sequences listed in the Global Initiative on Sharing All Influenza Data (GISAID) EpiFlu Database [16]. Details on the GISAID sequences used (including isolate IDs, names, originating lab) are listed in Additional file 3. Using tools available on the NIAID Influenza Research Database (IRD; http://www.fludb.org) [17], phylogenetic trees were built using PhyML [18] and IRD-defined settings (HKY model). Trees were visualized with FigTree 1.4.3. HA sequences from this study are available on GenBank (accession numbers MT077126-MT077135 and MT081183-MT081193).

### Statistical analysis

Following the GIHSN protocol, we chose a convenience sample of a minimum of 100 laboratory-confirmed influenza cases [11]. The study investigators estimated that there would be 2116 to 2260 eligible patients during the year at the three participating sites. Based on previous epidemiological studies conducted in Southeast Asia [7] and investigators experience, and in order to meet the expected number of laboratory confirmed influenza cases, assuming a conservative influenza positivity rate of 7%, a minimum of 1429 samples would be required. Based on monthly estimates of the numbers of ILI patients visiting each individual site and allowing up to 5.5% over-sampling, a sample size of 1508 subjects was defined, with an estimated average number of five patients per week at the University of Malaya Medical Centre and 12 patients per week each at Kuala Lumpur General Hospital and Selayang Hospital to be enrolled.

Influenza confirmation rates, and the distribution of the different influenza strains (A/H1N1, A/H3N2, B/Yamagata, B/Victoria) among confirmed cases, were assessed per week and per month for all included patients and presented as numbers and percentages, along with numbers enrolled. The proportion of total laboratory samples that tested positive for influenza virus was calculated as a 4-weekly moving average to smooth out the curves. This was done as some weekly numbers were low, and individual weekly rates could be disproportionately impacted by small differences in influenza detections. The 4-weekly moving average influenza positive rate for each epidemiological week (EW) = (total number of influenza cases in the last 4 weeks)/(total number of cases in the last 4 weeks). The exception was for the first datapoint after the third week of the study, which used the average for the first 3 weeks.

Patient characteristics at enrolment were described in terms of frequency and mean, overall, by influenza status and by age group (≥65 and < 65 years; and 18–24, 25–34, 35–44, 45–54, 55–64, 65–74, ≥75 years). The demographic and clinical characteristics of the two groups (i.e., influenza positive and influenza negative) were compared using the Chi-square test or the two-sided Fisher’s exact test (when any category had *n* < 5). All variables were tested for level one interactions.

To assess potential predictors of influenza positivity, crude odds ratios (ORs) and their 95% confidence intervals (95% CIs) were calculated with univariate logistic regression; variables significant at *p* value ≤0.25 were retained for inclusion in a multivariate logistic model. Multivariate analysis was conducted through a backward logistic regression with corresponding adjusted ORs and 95% CIs calculated; only variables significant at *p* value < 0.05 were kept in the final model.

Exploratory analyses were conducted to describe clinical outcomes (blood pressure on admission, number of days hospitalized, ICU or HDU admission and reasons, deaths) by influenza status. To assess predictors (sociodemographic and health history parameters) of severe outcomes in the study population, logistic regression was performed as described above. “Age”, “gender” and “presence of comorbidities” were forced-in covariates in the multivariate model.

Statistical analyses were conducted using SPSS Statistics version 22 (IBM, USA).

## Results

### Patients

**Fig. 1:**
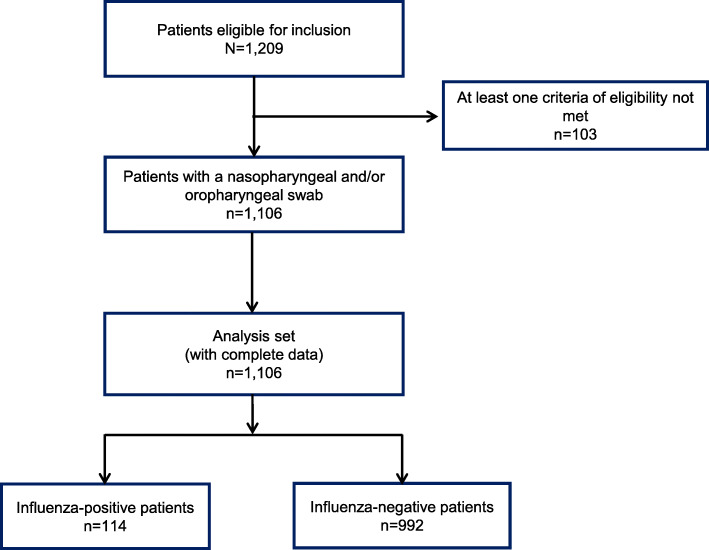
Participant flow through the study

Of 1209 patients assessed, 1106 met study inclusion criteria and provided nasopharyngeal and/or oropharyngeal swabs and were thus included in the analysis set (Fig. 1): 300 participants from the University of Malaya Medical Centre, 303 from the Kuala Lumpur General Hospital and 503 from the Selayang Hospital.

Characteristics of the patients enrolled are summarized in Table 1. The mean (SD) age of participants included in the analysis set was 59.6 (17.6) years; 54.1% of patients were aged < 65 years and 50.2% of patients were women; the majority of patients had at least 1 comorbidity (89.9%) and were not current or ex-smokers (59.9%). Most (75.8%) patients had a diagnosis upon admission to hospital that included CAP, 21.6% had AECOPD and 29.6% had AEBA; the most common comorbidities were cardiovascular disease (49.2%) and diabetes (36.2%). Only 2.0% had received influenza vaccination within the past 12 months. Due to the low rate of vaccination and the concern with statistical power and limited interpretation, vaccination status was excluded from subsequent analysis.

**Table 1:** Patients characteristics at enrolment

	Influenza-positive (*N* = 114)	Influenza-negative (*N* = 992)	Total (*N* = 1106)	χ^2^(df) Or t (df)	*p*-value^#^
Enrolment site, n (%)					
HKL	28 (24.6%)	275 (27.7%)	303 (27.4%)	1.495 (2)	0.474
UMMC	28 (24.6%)	272 (27.4%)	300 (27.1%)		
UiTM	58 (50.8%)	445 (44.9%)	503 (45.5%)		
Age, years					
Median (min; max)	64 (18; 97)	63 (18; 101)	63 (18; 101)	N/A	N/A
Mean (SD)	59.1 (18.1)	59.6 (17.6)	59.6 (17.6)	0.315 (1104)	0.753
Age group, years					
18–24	40 (4.0%)	5 (4.4%)	45 (4.1%)	2.576 (6)	0.860
25–34	75 (7.6%)	12 (10.5%)	87 (7.9%)		
35–44	94 (9.5%)	12 (10.5%)	106 (9.6%)		
45–54	118 (11.9%)	12 (10.5%)	130 (11.8%)		
55–64	211 (21.3%)	19 (16.7%)	230 (20.8%)		
65–74	256 (25.8%)	31 (27.2%)	287 (25.9%)		
≥ 75	198 (20%)	23 (20.2%)	221 (20.0%)		
Gender					
Male	46 (40.4%)	505 (50.9%)	551 (49.8%)	4.558 (1)	0.033*
Female	68 (59.6%)	487 (49.1%)	555 (50.2%)		
Mean number of comorbidities (SD)	1.57 (1.20)	1.67 (1.01)	1.66 (1.03)	0.982 (1104)	0.326
Significant comorbidities^†^					
No	20 (17.5%)	92 (9.3%)	112 (10.1%)	7.683 (1)	0.006*
Yes	94 (82.5%)	900 (90.7%)	994 (89.9%)		
Type of comorbidities					
Cardiovascular disease					
No	66 (57.8%)	496 (50%)	562 (50.8%)	2.550 (1)	0.110
Yes	48 (42.1%)	496 (50%)	544 (49.2%)		
Diabetes					
No	70 (61.4%)	636 (64.1%)	706 (63.8%)	0.325 (1)	0.569
Yes	44 (38.6%)	356 (35.9%)	400 (36.2%)		
Renal impairment					
No	100 (87.7%)	853 (86%)	953 (86.2%)	0.257 (1)	0.612
Yes	14 (12.3%)	139 (14%)	153 (13.8%)		
Autoimmune disease					
No	105 (92.1%)	941 (94.9%)	1046 (94.6%)	1.511 (1)	0.219
Yes	9 (7.9%)	51 (5.1%)	60 (5.4%)		
Asthma					
No	79 (69.3%)	699 (70.5%)	778 (70.3%)	0.067 (1)	0.796
Yes	35 (30.7%)	293 (29.5%)	328 (29.7%)		
COPD					
No	93 (81.6%)	766 (77.2%)	859 (77.7%)	1.121 (1)	0.290
Yes	21 (18.4%)	226 (22.8%)	247 (22.3%)		
Other (Liver cirrhosis, neurological disorder, HIV, chronic lung disease and malignancy)					
No	107 (93.9%)	900 (90.7%)	1007 (91.0%)	1.232 (1)	0.267
Yes	7 (6.1%)	92 (9.3%)	99 (9.0%)		
BMI mean (SD)	26.4 (7.5)	26.1 (6.9)	26.5 (7.6)	0.462 (632)	0.670
Smoking status					
Never	78 (68.4%)	584 (59.2%)	662 (59.9%)	4.508 (2)	0.105
Former	21 (18.4%)	199 (20.2%)	220 (19.9%)		
Current	15 (13.2%)	204 (20.7%)	219 (19.8%)		
Missing	NA	NA	5 (0.5%)		
Socio-professional category‡					
High socio-professional category	16 (14%)	117 (11.8%)	133 (12%)	3.340 (4)	0.503
Middle socio-professional category	15 (13.2%)	112 (11.3%)	127 (11.5%)		
Low socio-professional category	5 (4.4%)	85 (8.6%)	90 (8.1%)		
Not working/unknown	61 (53.5%)	508 (51.2%)	569 (51.4%)		
Retired	17 (14.9%)	170 (17.1%)	187 (16.9%)		
Diagnoses at admission					
Acute exacerbation of asthma (AEBA)					
No	79 (69.3%)	700 (70.6%)	779 (70.4%)	0.079 (1)	0.779
Yes	35 (30.7%)	292 (29.4%)	327 (29.6%)		
Acute exacerbation of COPD (AECOPD)					
No	95 (83.3%)	772 (77.8%)	867 (78.4%)	1.833 (1)	0.176
Yes	19 (16.7%)	220 (22.2%)	239 (21.6%)		
Community-acquired pneumonia (CAP)					
No	26 (22.8%)	242 (24.4%)	268 (24.2%)	0.140 (1)	0.708
Yes	88 (77.2%)	750 (75.6%)	838 (75.8%)		
Systemic symptoms at admission					
At least 1 systemic symptoms present					
No	0 (0%)	3 (0.3%)	3 (0.3%)		1.000^¥^
Yes	114 (100%)	989 (99.7%)	1103 (99.7%)		
Fever					
No	21 (18.4%)	293 (29.5%)	314 (28.4%)	6.214 (1)	0.013*
Yes	93 (81.6%)	699 (70.5%)	792 (71.6%)		
Headache					
No	97 (85.1%)	842 (84.9%)	939 (84.9%)	0.003 (1)	0.953
Yes	17 (14.9%)	150 (15.1%)	167 (15.1%)		
Malaise					
No	66 (57.9%)	492 (49.5%)	558 (50.5%)	2.816 (1)	0.093
Yes	48 (42.1%)	500 (50.4%)	548 (49.5%)		
Myalgia					
No	95 (83.3%)	838 (84.5%)	933 (84.4%)	0.101 (1)	0.750
Yes	19 (16.7%)	154 (15.5%)	173 (15.6%)		
Respiratory symptoms					
At least 1 respiratory symptoms present					
No	0 (0%)	1 (0.1%)	1 (0.1%)		1.000^¥^
Yes	114 (100%)	991 (99.9%)	1105 (99.9%)		
Cough					
No	11 (9.6%)	82 (8.3%)	93 (8.4%)	0.254 (1)	0.614
Yes	103 (90.4%)	910 (91.7%)	1013 (91.6%)		
Dyspnea					
No	30 (26.3%)	143 (14.4%)	173 (15.6%)	10.974 (1)	0.001*
Yes	84 (73.7%)	849 (85.6%)	933 (84.4%)		
Sore throat					
No	93 (81.6%)	805 (81.1%)	898 (81.2%)	0.012 (1)	0.911
Yes	21 (18.4%)	187 (18.9%)	208 (18.8%)		
Enrolment month					
July 2018	4 (3.5%)	32 (3.2%)	36 (3.3%)	29.843 (13)	0.005*
August 2018	5 (4.4%)	56 (5.6%)	61 (5.5%)		
September 2018	1 (0.9%)	22 (2.2%)	23 (2.1%)		
October 2018	4 (3.5%)	33 (3.3%)	37 (3.3%)		
November 2018	14 (12.3%)	40 (4%)	54 (4.9%)		
December 2018	7 (6.1%)	29 (2.9%)	36 (3.3%)		
January 2019	6 (5.3%)	63 (6.4%)	69 (6.2%)		
February 2019	14 (12.3%)	81 (8.2%)	95 (8.6%)		
March 2019	5 (4.4%)	63 (6.4%)	68 (6.1%)		
April 2019	7 (6.1%)	85 (8.6%)	92 (8.3%)		
May 2019	8 (7.0%)	120 (12.1%)	128 (11.6%)		
June 2019	18 (15.8%)	180 (18.1%)	198 (17.9%)		
July 2019	20 (17.5%)	137 (13.8%)	157 (14.2%)		
August 2019	1 (0.9%)	51 (5.1%)	52 (4.7%)		
≥1 hospitalization within the past 12 months					
Yes	29 (25.4%)	343 (34.6%)	372 (33.6%)	3.825 (1)	0.050
No	85 (74.6%)	649 (65.4%)	734 (66.4%)		
≥1 consultation within the past 3 months					
Yes	32 (28.1%)	352 (35.5%)	384 (34.7%)	2.480 (1)	0.115
No	82 (71.9%)	640 (64.5%)	734 (66.4%)		
Flu Vaccination within past 12 months and more than 14 days					
Yes	2 (1.8%)	20 (2%)	22 (2%)	0.036 (1)	0.850^¥^
No or unclear	112 (98.2%)	972 (98%)	1084 (98%)		

†Comorbidities that involve organ dysfunction are considered as significant comorbidities; ‡Socio-professional categories are defined in Additional file 1; ^#^Chi-square test (unless otherwise indicated); ^¥^Fisher’s exact test; *p-value < 0.05

HKL, Kuala Lumpur General Hospital; NA, not available; UMMC, University of Malaya Medical Centre; UiTM, Selayang Hospital

### Influenza positivity rate

Overall, 10.3% (114/1106) of included patients were positive for influenza during the study, with similar positivity rates observed across the three sites (range, 9.2–11.5%).

**Fig. 2:**
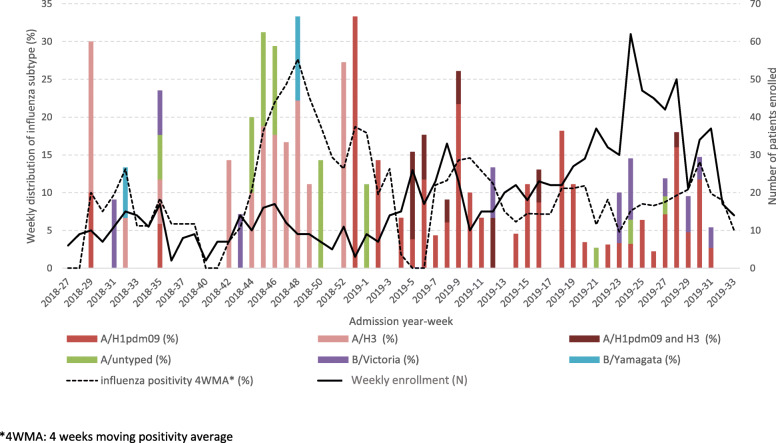
Number of patients enrolled and influenza-positive rate per week of hospital admission overall and by subtypes. Influenza-positive rate and proportions of circulating virus subtypes in the three study sites, July 2018 to August 2019. The influenza-positive rate shown for each epidemiological week is the 4-week moving average (MA; dashed line), which is the average rate of that week and the preceding 3 weeks. 4WMA: 4 weeks moving positivity average

Over the study period, most influenza cases were influenza A (85.1%); the dominant strain was A/H1N1pdm09 (57.0% of all influenza-confirmed cases, including 7.9% co-infection with A/H3N2). Over the first several weeks of the study, July through August 2018, all influenza subtypes were present and accounted for similar proportions of cases (Fig. 2). Following a 7-week period (2 September–21 October 2018), during which limited influenza activity was detected, there was a spike in the influenza positive rate in November 2018 coinciding with A/H3N2 becoming the predominant circulating strain for about 2 months. A/H1N1pdm09 emerged as the main circulating strain from January 2019, with an associated increase in monthly influenza positive rate observed from February, and remained predominant until the end of the study in August 2019. There was a brief increase in B/Victoria positive rates, around June 2019 and in the proportion of total influenza positive cases in June and July 2019, coinciding with an increase in monthly patient enrolment (> 100/month) from May through August 2019 (Fig. 2).

### Phylogenetic analysis of influenza sequences

A total of 11 A/H1N1pdm09 and 10 A/H3N2 sequences were generated and compared to contemporaneous and reference strains. The phylogenetic tree of A/H1N1pdm09 (Additional file 4A) showed that all 11 sequences from this study were from the subclade 6b1.A183P5. Of the 10 A/H3N2 virus sequences from this study, 6 were in the 3C.2a1b + 131 K subclade, 3 in the 3C.2a1b + 135 K subclade, and 1 in the 3C.2a3 subclade (Additional file 4B).

### Variables associated with influenza positivity and severe outcome

The variables associated with influenza positivity (*p* ≤ 0.25) in univariate analysis included (Table 2): gender (female), smoker, hospitalization over the previous 12 months, consultations over the previous 3 months, diagnosis of AECOPD on admission, presence of significant comorbidities, and the presence of fever, malaise or dyspnea. The variables that remained associated with influenza positivity in multivariate analyses were absence of significant comorbidities (none versus any comorbidity [OR (95%CI), 0.565 (0.329–0.970)], *p* = 0.038) and dyspnea (0.544 (0.341–0.868)], *p* = 0.011) (Table 2).

**Table 2:** Risk variables associated with influenza positivity

Variables	Univariate analysis		Multivariate analysis	
	OR (CI 95%)	***P***-value	OR (CI 95%)	***P***-value
Age				
< 65 years	Ref	Ref	Ref	Ref
≥ 65 years	1.067 (0.723–1.573)	0.745	1.072 (0.710–1.618)	0.741
**Gender**				
**Male**	**Ref**	**Ref**	**Ref**	**Ref**
**Female**	**1.533 (1.033–2.274)**	0.034*	**1.465 (0.982–2.184)**	**0.061**
Smoking status				
Non-smoker	Ref	Ref		
Ex-smoker	0.790 (0.475–1.313)	0.363		
Smoker	0.551 (0.310–0.978)	0.042		
BMI classification (Asian)				
Normal (18.5–24.9)	Ref	Ref		
Obese (≥25.0)	1.136 (0.651–1.983)	0.653		
Underweight (< 18.5)	1.603 (0.737–3.487)	0.234		
Socio-professional category†				
High	Ref	Ref		
Middle	0.979 (0.462–2.074)	0.957		
Low	0.430 (0.152–1.220)	0.430		
Unemployed/unknown	0.878 (0.489–1.578)	0.664		
Retired	0.731 (0.355–1.506)	0.731		
Hospitalized within the past 12 months				
No	Ref	Ref	Ref	Ref
Yes	0.646 (0.415–1.004)	0.050	0.808 (0.507–1.288)	0.370
Consultations within the past 3 months				
No	Ref	Ref	Ref	Ref
Yes	0.710 (0.462–1.089)	0.115	0.745 (0.483–1.151)	0.185
Diagnosis on admission				
AECOPD				
No	Ref	Ref	Ref	Ref
Yes	0.702 (0.419–1.174)	0.176	1.314 (0.688–2.511)	0.408
AEBA				
No	Ref	Ref		
Yes	1.062 (0.697–1.618)	0.779		
CAP				
No	Ref	Ref		
Yes	1.092 (0.689–1.731)	0.708		
**Presence of significant comorbidities‡**				
**No**	**Ref**	**Ref**	**Ref**	**Ref**
**Yes**	**0.480 (0.283–0.815)**	0.006*	**0.565 (0.329–0.970)**	0.038*
Temperature on presentation (≥38 °C)				
No	Ref	Ref		
Yes	1.415 (0.873–2.292)	0.159		
Systemic symptoms				
**Fever**				
**No**	**Ref**	**Ref**	**Ref**	**Ref**
**Yes**	**1.856 (1.134–3.039)**	0.013*	**1.544 (0.932–2557)**	**0.091**
Headache				
No	Ref	Ref		
Yes	0.984 (0.571–1.695)	0.953		
Malaise				
No	Ref	Ref	Ref	Ref
Yes	0.716 (0.484–1.059)	0.093	1.084 (0.674–1.742)	0.740
Myalgia				
No	Ref	Ref		
Yes	1.088 (0.646–1.834)	0.750		
Respiratory symptoms				
Cough				
No	Ref	Ref		
Yes	0.844 (0.435–1.635)	0.614		
**Dyspnea**				
**No**	**Ref**	**Ref**	**Ref**	**Ref**
**Yes**	**0.472 (0.300–0.742)**	0.001*	**0.544 (0.341–0.868)**	0.011*
Sore throat				
No	Ref	Ref		
Yes	0.972 (0.590–1.602)	0.972		
Vaccination within the past 12 months				
No or unclear history	Ref	Ref		
Yes	0.554 (0.131–2.344)	0.422		

Bold = variables left in final model

*p-value < 0.05; †Socio-professional categories are defined in Additional file 1; ^‡^Comorbidities that involve organ dysfunction are considered as significant comorbidities

Among patients with influenza infection, 14% experienced at least 1 severe outcome, 13.2% were admitted to ICU/HDU and 2.6% died. Exploratory analyses of hospitalization outcomes according to influenza status are presented in Table 3. Among patients admitted to the ICU or HDU (*n* = 184), septicemic shock was a more likely cause among those who were influenza-positive than those who were influenza-negative.

**Table 3:** Exploratory data on hospitalization outcomes according to influenza status

Variables	Total (***N*** = 1106)	Influenza positive (***N*** = 114)	Influenza negative (***N*** = 992)	Univariate analysis			
				OR (95% CI)	***P***-value	Adjusted OR (95% CI)^a^	***P***-value
No. of days in hospital (*n* = 1097)							
Mean no. of hospital admission (days)	7.12 ± 8.059	6.89 ± 7.707 (*n* = 114)	7.15 ± 8.102 (*n* = 983)				
Prolonged hospitalization (≥6 days)	486 (44.3%)	43 (37.7%)	443 (45.1%)	0.736 (0.495–1.100)	0.136	0.766 (0.512–1.146)	0.194
Reason for ICU or HDU admission							
Not known	46 (25%)	3 (20%)	43 (25.4%)	0.218 (1)	0.641	0.805 (0.275–2.359)	0.692
Known	138 (12.5%)	12 (80%)	126 (74.6%)				
Respiratory failure	119 (64.7%)	10 (66.7%)	109 (64.5%)	1.101 (0.360–3.370)	0.866	1.087 (0.348–3.402)	0.886
**Septicemic shock**	**27 (14.7%)**	**5 (33.3%)**	**22 (13%)**	**3.341 (1.044–10.692)**	0.042*	**3.957 (1.171–13.376)**	0.027*
Respiratory failure and septicemic shock	5 (2.7%)	1 (6.7%)	4 (2.4%)	2.946 (0.308–28.185)	0.348	4.925 (0.448–54.017)	0.192
ICU or HDU admissions							
No	922 (83.4%)	99 (86.8%)	823 (83%)	Ref	Ref	Ref	Ref
Yes	184 (16.6%)	15 (13.2%)	169 (17%)	0.738 (0.418–1.302)	0.294	0.785 (0.443–1.392)	0.408
Death in hospital							
No	1080 (97.6%)	111 (97.4%)	969 (97.7%)	Ref	Ref	Ref	Ref
Yes	26 (2.4%)	3 (2.6%)	23 (2.3%)	1.139 (0.336–3.853)	0.835	1.138 (0.333–3.892)	0.836
Severe outcome (death in hospital or ICU/HDU admission without death)							
No	912 (82.5%)	98 (86%)	814 (82.1%)	Ref	Ref	Ref	Ref
Yes	194 (17.5%)	16 (14%)	178 (17.9%)	0.747 (0.430–1.298)	0.299	0.792 (0.453–1.384)	0.413

^a^Adjusted for age, gender and presence of comorbidities

## Discussion

Influenza was detected in 10.3% of patients presenting with CAP, AECOPD and/or AEBA who had onset of ILI ≤10 days prior to admission in our study. In multivariate analyses, the absence of significant comorbidities and absence of dyspnea at admission were independent predictors for influenza infection.

The influenza positivity rate in this study is within the range of influenza detection rates previously described (rates of up to 5–14%) among adults hospitalized with severe acute respiratory symptoms in countries in East and Southeast Asia [6, 7]. While influenza is typically present year-round in tropical and subtropical regions, available data have shown peaks of influenza activity occurring earlier and/or later in the year, depending on the country [8, 19–22]. Accordingly, reports of the presence or absence of seasonal peaks in Malaysia have been inconsistent [8, 19, 21]. Observations based on laboratory surveillance between 2011 and 2016 demonstrated variable periods of higher transmission coinciding with winter seasons of northern (November–February) and/or southern (July–September) hemisphere regions [23].

In our study, a higher rate of influenza-positive patients was observed between November 2018 and February 2019, and June to July 2019. Influenza positivity peaked in June and July 2019, coinciding with increased enrolment of participants from May through July, possibly due to increased circulating influenza during this time. The two periods of increased influenza activity observed in our study broadly correspond to the seasons occurring in the Northern and Southern hemispheres, respectively, in-line with previous observations [23]. Similar trends were also observed among the numbers of Malaysian isolates sent to the WHO Collaborating Centre for Reference and Research on Influenza in Melbourne in 2018–2019 [24].

Notably, the dominant serotype among confirmed cases in our study differed between the first (A/H3N2; July through December 2018) and second half (A/H1N1pdm09; January through August 2019) of the study. Increases in transmission rates may be associated with changes in the predominant circulating influenza virus type or subtype [23, 25], likely due to a relative lack of population immunity to newly emergent viruses. A spike in influenza positivity in November 2018 coincided with increased circulation of A/H3N2 (and detection of B/Yamagata) relative to other strains detected, the February 2019 influenza positivity spike coincided with an increase circulation of A(H1N1)pdm09 following its emergence in January 2019, and the increased positivity rate in June–July 2019 coincided with greater proportions of influenza B/Victoria detected.

Phylogenetic analyses in this study show that Malaysian A/H1N1pdm09 and A/H3N2 viruses from 2018 to 2019 were heterogeneous, falling into numerous different subclades. All 11 A/H1N1pdm09 sequences from this study, and contemporaneous sequences isolated separately in 2019, were from the subclade 6b1.A183P5, while earlier Malaysian sequences from early-mid 2018 were from 6b1.A183P4 and 6b1.A183P6 and some from late 2018 belonged to the 6b1.A183P2 subclade. Of the 10 A/H3N2 virus sequences from this study, six were in the 3C.2a1b + 131 K subclade, three in the 3C.2a1b + 135 K subclade, and one in the 3C.2a3 subclade; the same subclades have been identified for other Malaysian A/H3N2 virus sequences detected in late 2018 and early 2019, with a single sequence in the 3C.2a1b + 135 N subclade.

While our study population had a high rate of comorbidities, those without significant comorbidities were more likely to be influenza-positive. Virulent pathogens like influenza virus are more likely to account for a higher proportion of infectious causes in those hospitalized without underlying comorbidities, while those with comorbidities may be at greater risk of hospitalization due to a wider range of respiratory pathogens, such that other pathogens such as rhinovirus make a larger contribution [26]. In addition, individuals categorized as having no significant comorbidities (i.e. excluding those with COPD and asthma) were by definition all enrolled with a diagnosis of CAP for which influenza is a commonly identified pathogen.

Previous studies have generally found older age and certain comorbidities to be associated with severe influenza outcomes, although the definitions of risk factors and the populations studied have been variable. A study of hospitalized adults with COPD found older age (> 75 years), comorbidities of heart disease, home oxygen use and diabetes with end-organ complications, and current smoking as risk factors for influenza-related severe outcomes (30-day mortality or ICU admission) [27]. In another study, age ≥ 65 years and comorbidities of diabetes and acute kidney injury were associated with severity of influenza-associated pneumonia [28]. In a prior systematic review and meta-analysis, older age, morbid obesity (adjusted for cardiovascular comorbidities and diabetes) and chronic illness (immunosuppression, cardiovascular disease, chronic lung disease, neuromuscular disease, neurological disease, chronic renal disease, and metabolic diseases), but not sex, were associated with an elevated risk of death from influenza; however, the authors concluded that the overall level of evidence was low and that more rigorous studies were needed. In our specific study population of patients hospitalized for CAP, AECOPD and/or AEBA, just under half were aged ≥65 years, suggesting that younger adults are also at risk of ILI-associated hospitalizations, whether associated with confirmed influenza or not. Influenza status was not a predictor of severe outcomes, ICU/HDU admissions or deaths. However, it was associated with higher rates of sepsis in ICU/HDU admitted patients.

A number of limitations of our study should be considered. As we did not test for the presence of other respiratory viruses or bacteria, we cannot rule out the possibility that other pathogens may have had an effect on clinical outcome for these patients. Our study was conducted during an H1N1-dominant influenza season, which may also have had an impact on the findings from this study. Indeed, it is generally accepted that there may be a higher disease burden (hospitalizations and deaths) among older adults during H3N2-dominant influenza seasons, while influenza A H1N1pdm09 has a lower attack rate with greater impact on younger adults [29–31]. Additional data are needed to better describe the associated burden of influenza infection associated with different dominant circulating influenza subtypes. While our study was based on a single year of surveillance data and limited to one area of Malaysia, it should be noted that there may be significant area-to-area and year-to-year variation. Additionally, the patients included in the current study had very low rates of influenza vaccination (3% overall; 1.8% in influenza-positive patients and 3.1% in influenza-negative patients), in-line with previous observations that vaccination uptake is generally low in Malaysia [8, 32], thus no conclusions can be drawn on the effect of influenza vaccination on our results. This study did not allow us to determine causality of variables associated with influenza positivity. Finally, the small sample size of influenza-positive patients as well as patients with severe outcomes prevented us from conducting further analysis of variables and predictors of severe influenza outcome.

To our knowledge, this is the first active surveillance study to look at severe influenza in adult hospitalized patients in Malaysia. We report that absence of significant comorbidities (versus any significant comorbidity) and absence of dyspnea were predictors of influenza positivity in hospitalized adults with CAP, AECOPD and/or AEBA, and describe very low rates of influenza vaccination even in patients with underlying comorbidities. These results show the contribution of influenza to hospitalization for severe respiratory illness and also documents the risk for a population not considered at-risk, adults without comorbidities. This will help raise awareness on influenza disease burden and the associated severe illness in the Malaysian population and help guide decisions on optimal implementation of prevention measures such as vaccination.

## Supplementary Information

**Additional file 1.** Socio-professional categories.

**Additional file 2.** Primers and probes used for detection, subtyping and sequencing influenza virus*.

**Additional file 3.** List of influenza sequences used in analysis.

**Additional file 4.** Phylogenetic trees of A/H1pdm and A/H3 sequences.

## Data Availability

The data that supports the findings of this study are available in the supplementary material of this article. HA sequences from this study are available on GenBank (accession numbers MT077126-MT077135 and MT081183-MT081193). Permanent link to sequencing data available at https://www.ncbi.nlm.nih.gov/nuccore/?term=MT077126%3AMT077135%5Baccn%5D+OR+MT081183%3AMT081193%5Baccn%5D.
