# Supplementary material for: Influenza in Malaysian adult patients hospitalized with community-acquired pneumonia, acute exacerbation of chronic obstructive pulmonary disease or asthma: a multicenter, active surveillance study
Source: BMC Infect Dis. 2021 Jul 5;21:644. doi: 10.1186/s12879-021-06360-9 (PMC8256617; doi:10.1186/s12879-021-06360-9)
Supplement: Supplementary file 1 — Additional file 1. Socio-professional categories. [file 12879_2021_6360_MOESM1_ESM.docx]

**Additional file 1: Socio-professional categories**

The socio-professional categories are defined as follow:

1. Executives of the public administration and companies with 10 or more employees. Professions with a graduate (MD, lawyers, architects) or a postgraduate degree (PhD).
2. Managers of firms with fewer than 10 employees. Professions associated with a first cycle university degree (diploma). Technicians. Artists. Athletes.
3. Administrative employees and professionals that give administrative support or financial management (accountants). Personal-services (i.e. hairstylist). Security. Self-employed. Supervisors.
4. Skilled manual workers (Specific training needed).
5. Semiskilled manual workers (No specific training but working on specific general fields giving assistance to the skilled manual workers: industry, building, furniture, fishing).
6. Unskilled workers.
7. Not classifiable/don’t know

For analysis purposes they were grouped as:

- Category 1-3 high socio-professional category
- Category 4-5 middle socio-professional category
- Category 6 low socio-professional category
- Category 7 unclassifiable
