# Supplementary material for: Influenza in Malaysian adult patients hospitalized with community-acquired pneumonia, acute exacerbation of chronic obstructive pulmonary disease or asthma: a multicenter, active surveillance study
Source: BMC Infect Dis. 2021 Jul 5;21:644. doi: 10.1186/s12879-021-06360-9 (PMC8256617; doi:10.1186/s12879-021-06360-9)
Supplement: Supplementary file 2 — Additional file 2. Primers and probes used for detection, subtyping and sequencing influenza virus*. [file 12879_2021_6360_MOESM2_ESM.docx]

**Additional file 2. Primers and probes used for detection, subtyping and sequencing influenza virus***

|  | | **Target gene** | **Sequence** | **Conc. (µM)** | **Product size (bp)** |
| --- | --- | --- | --- | --- | --- |
| **Influenza detection** | A | M | Forward: (FLUAM-7-F) 5’CTTCTAACCGAGGTCGAAACGTA-3’  Reverse: (FLUAM-161-R) 5’GGTGACAGGATTGGTCTTGTCTTTA-3’  Probe: (FLUAM-49-P6) 5’(FAM) –TCAGGCCCCCTCAAAGCCGAG-3’ (3IABkFQ) | 0.1  0.1  0.1 | 155 |
|  | B | HA | Forward: (FLUBHA-940-F) 5’AAATACGGTGGATTAAACAAAAGCAA-3’  Reverse: (FLUBHA-1109-R) 5’CCAGCAATAGCTCCGAAGAAA-3’  Probe: (FLUBHA-994-P4) 5’(JOE) –CACCCATATTGGGCAATTTCCTATGGC-3’ (3IABkFQ) | 0.1  0.1  0.1 | 170 |
| **Influenza A subtyping** | A/H1pdm | HA | Forward: (H1pdm-169-F) 5’AAACTATGCAAACTAAGAGGGGT-3’  Reverse: (H1pdm-297-R) 5’TGTTTCCACAATGTAGGACCA-3’  Probe: (H1pdm-244-P) 5’(JOE) – CCAGAGTGTGAATCACTCTCCACA-3’ (3IABkFQ) | 0.25  0.25  0.1 | 129 |
|  | A/H3 | HA | Forward: (H3-266-F) 5’ACCCTCAGTGTGATGGCTTTCAAA-3’  Reverse: (H3-373-R) 5’TAAGGGAGGCATAATCCGGCACA-3’  Probe: (H3-315-P) 5’(FAM) – ACGAAGCAAAGCCTACAGCAA CTGTT-3’ (3IABkFQ) | 0.25  0.25  0.1 | 108 |
| **Influenza B subtyping** | Victoria lineage | HA | Forward: (BHA-188F) 5’AGACCAGAGGGAAACTATGCCC-3’  Reverse: (BHA-270R) 5’TCCGGATGTAACAGGTCTGACTT-3’  Probe: (Probe-VIC2) 5’ (JOE) – CAGACCAAAATGCACGGGGAAHATACC-3’ (3IABkFQ)  Probe: (Probe-YAM2) 5’ (FAM) – CAGRCCAATGTGTGTGGGGAYCACACC-3’ (3IABkFQ) | 0.5  0.5  0.2  0.2 | 137 |
|  | Yamagata lineage | HA |  |  |  |
| **Influenza A sequencing** | A/H1pdm | HA | H1F1 (forward): 5’-AGCAAAAGCAGGGGAAAATAAAAGC-3’  H1R1264 (reverse): 5’-CCTACTGCTGTGAACTGTGTATTC-3’ | 0.4  0.4 | 1264 |
|  |  |  | H1F848 (forward): 5’-GCAATGGAAAGAAATGCTGGATCTG-3’  HARUc (forward): 5’-ATATCGTCTCGTATTAGTAGAAACAAGGGTGTTTT-3’ | 0.4  0.4 | 945 |
|  | A/H3 | HA | H3A1F6 (forward): 5’ AAGCAGGGGATAATTCTATTAACC-3’  H3A1R1 (reverse): 5’ GTCTATCATTCCCTCCCAACCATT-3’ | 0.4  0.4 | 1127 |
|  |  |  | H3A1F3 (forward): 5’ TGCATCACTCCAAATGGAAGCATT-3’  HARUc (reverse): 5’ ATATCGTCTCGTATTAGTAGAAACAAGGGTGTTTT-3’ | 0.4  0.4 | 863 |

*As described by the World Health Organization information for the molecular detection of influenza viruses (World Health Organization, 2017)

HA, hemagglutinin

**Supplementary reference**

World Health Organization (2017). WHO information for the molecular detection of influenza viruses. Available at: https://www.who.int/influenza/gisrs_laboratory/WHO_information_for_the_molecular_detection_of_influenza_viruses_20171023_Final.pdf?ua=1. Accessed 17 June 2020.
