# Supplementary material for: Influenza in Malaysian adult patients hospitalized with community-acquired pneumonia, acute exacerbation of chronic obstructive pulmonary disease or asthma: a multicenter, active surveillance study
Source: BMC Infect Dis. 2021 Jul 5;21:644. doi: 10.1186/s12879-021-06360-9 (PMC8256617; doi:10.1186/s12879-021-06360-9)

**Additional file 4.** Phylogenetic trees of A/H1pdm and A/H3 sequences. **A.** Phylogenetic tree of A/H1pdm sequences (1691 nucleotides) showing selected influenza strains detected in 2018-2019, and reference and vaccine strains (blue). Malaysian strain sequences from GISAID are in purple, and sequences from this study are in red. Individual sequences are identified as: sequence name/GISAID Epi accession number/date of specimen. Sequences from this study are identified as: sequence name/GenBank accession number/date of specimen. Subclades within clade 6b1.A are labelled and shown with differently colored branches. The scale bar represents the percentage nucleotide difference between sequences.


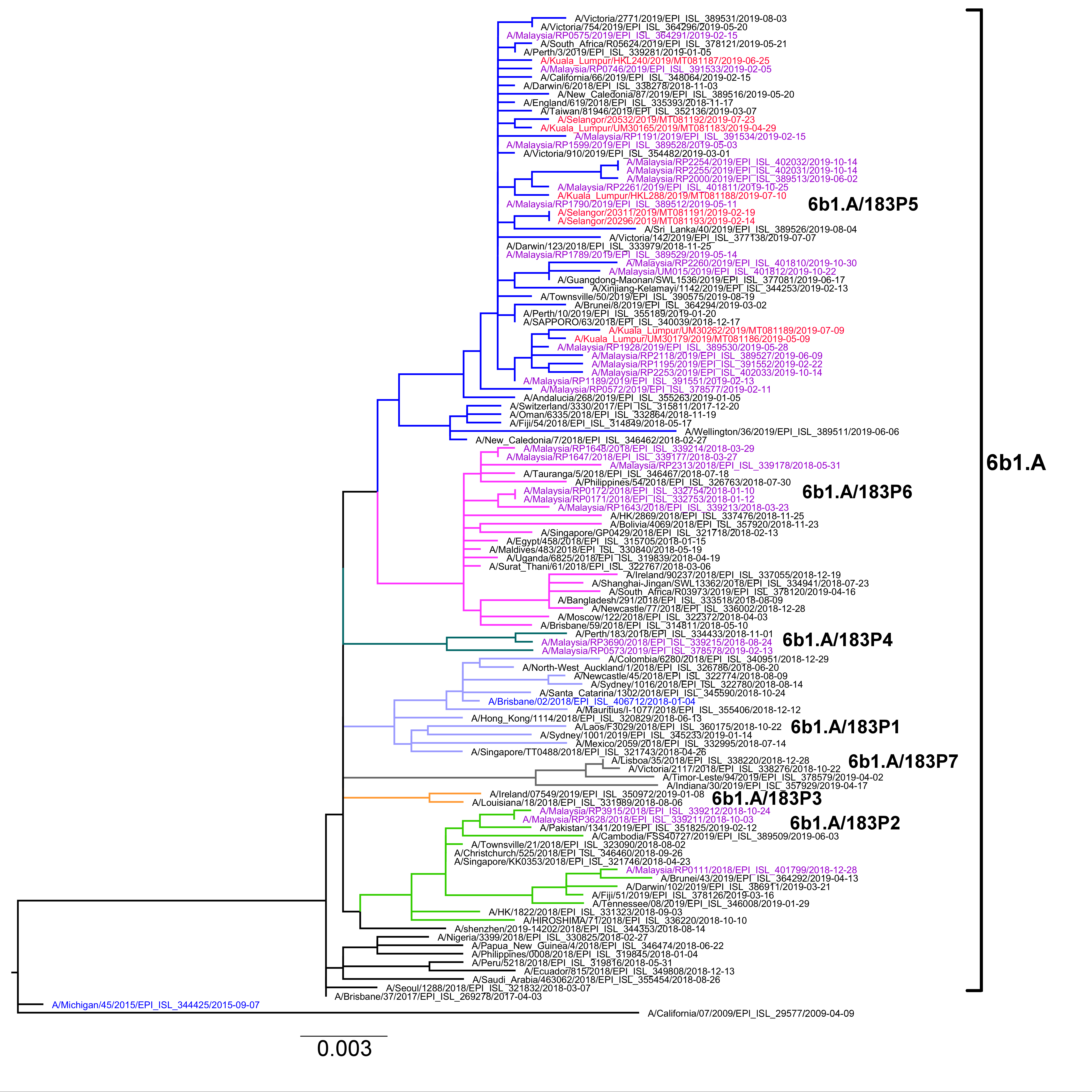


**B.** Phylogenetic tree of A/H3 sequences (1691 nucleotides) showing selected influenza strains detected in 2018-2019, and reference and vaccine strains (blue). Malaysian strain sequences from GISAID are in purple, and sequences from this study are in red. Individual sequences are identified as: sequence name/GISAID Epi accession number/date of specimen. Sequences from this study are identified as: sequence name/GenBank accession number/date of specimen. Subclades are labelled and shown with differently colored branches. The scale bar represents the percentage nucleotide difference between sequences.


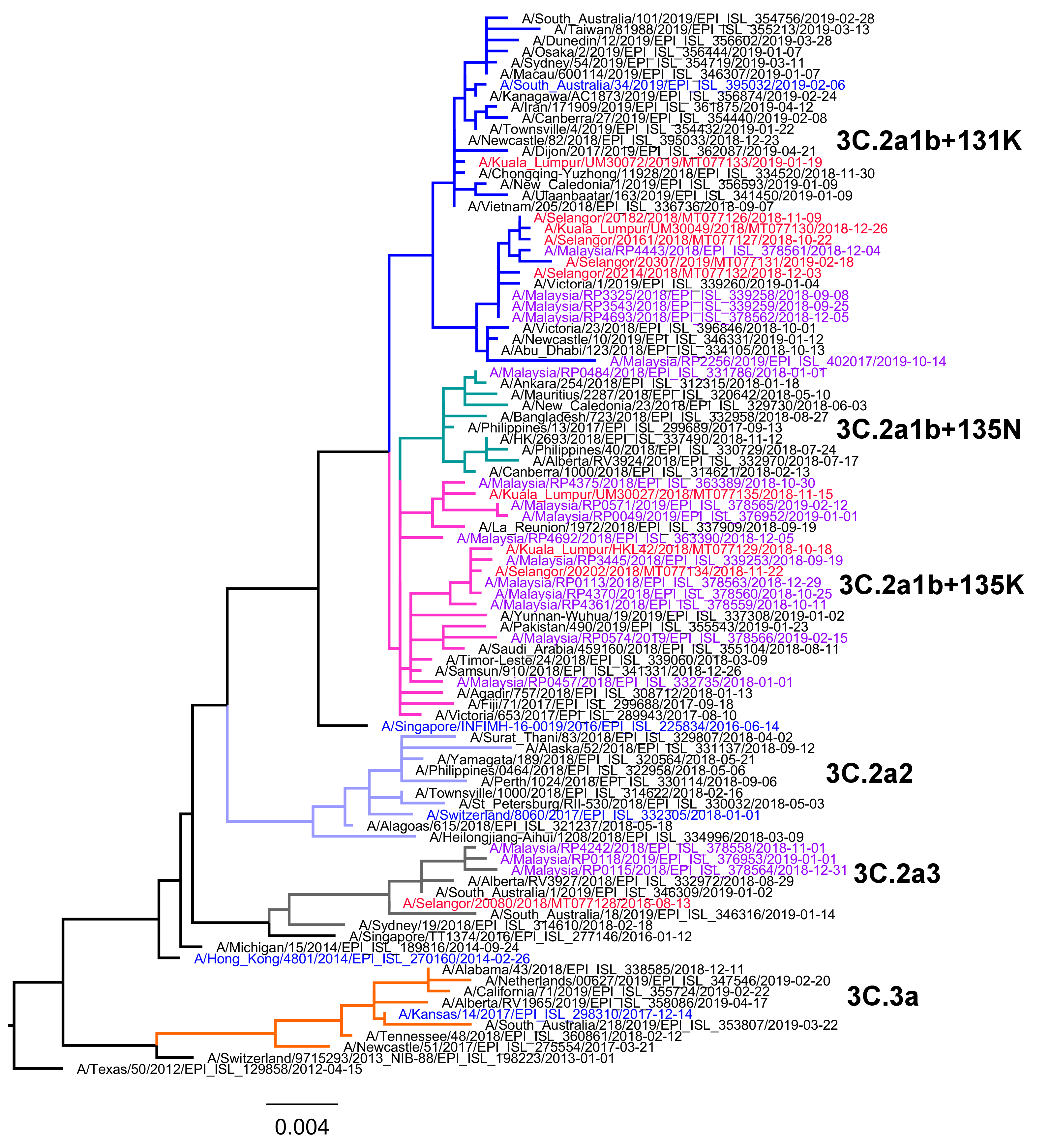

Supplement: Supplementary file 4 — Additional file 4. Phylogenetic trees of A/H1pdm and A/H3 sequences. [file 12879_2021_6360_MOESM4_ESM.docx]
